# Laparoscopic suture repair for perforated peptic ulcer disease: a meta-review and trial sequential analysis

**DOI:** 10.3389/fsurg.2025.1496192

**Published:** 2025-02-12

**Authors:** S. I. Panin, T. V. Nechay, I. V. Sazhin, K. Yu. Melnikov-Makarchuk, A. V. Sazhin, A. V. Puzikova, A. N. Akinchits, A. V. Bykov

**Affiliations:** ^1^ Volgograd State Medical University, Volgograd, Russia; ^2^ Pirogov Russian National Research Medical University, Moscow, Russia

**Keywords:** perforated peptic ulcer, overview of reviews, meta-epidemiology, umbrella review, trial sequential analysis

## Abstract

**Background:**

The number of systematic reviews (SRs) and meta-analyses in surgery is growing exponentially. Meta-epidemiology, as a form of evidence synthesis, allows for the pooling of data and assessment of the diversity present in multiple and overlapping SRs.

**Aim of the research:**

This study aimed to summarize evidence from systematic reviews of randomized controlled trials and reanalyze outcome data on laparoscopic suture repair of perforated peptic ulcers using trial sequential analysis (TSA).

**Materials and methods:**

The Cochrane Library, PubMed, Embase, CINAHL, eLibrary, and ClinicalTrials.gov were searched before 1 June 2024. A meta-epidemiological approach and TSA were used.

**Results:**

In total, 16 relevant Cochrane and non-Cochrane SRs that addressed laparoscopic repair of perforated peptic ulcers (PPUs) were identified and critically appraised. Three overlapping reviews of RCTs met the inclusion criteria. Their pooled results showed a lower postoperative pain score after laparoscopic repair compared with open closure on postoperative day 1 as the only significant outcome. There were no significant differences in other clinical outcomes. The re-analyses of meta-analytic findings and adjustments of sample size by TSA confirmed that laparoscopic repair was associated with less postoperative pain [100% of the diversity-adjusted required information size (DARIS) was reached]. The calculated DARIS for operative time and hospital stay were 40.1% and 14.6%, respectively, and the TSA showed neither significant benefit nor harm of laparoscopic surgery in the attained information size in the meta-analysis. Further trials with regard to mortality, surgical site infection, and intra-abdominal abscess are not very promising because the DARIS did not exceed 5% after combining the results of eight RCTs.

**Conclusion:**

Summarization of evidence from systematic reviews and reanalysis using TSA confirmed sufficient evidence for only one outcome, namely, that laparoscopic suture repair of PPUs is accompanied by lower pain scores at 24–72 h. Regarding the issues of postoperative complications and mortality, achieving DARIS through additional studies seems unpromising.

## Background

A perforated peptic ulcer (PPU) is a common disease that requires emergency surgery and is the cause of one-third of all peptic ulcer-related deaths (1, 2). Despite a decrease in the absolute number of admissions and surgeries performed, there has been an increase in the absolute number of in-hospital and postoperative mortality rates (2–4). Thus, in recent years, in-hospital mortality for PPUs in Russia has increased from 12.11% to 13.23% and postoperative mortality from 10.77% to 12.5% (3). This trend is consistent with data from other countries. According to observational studies, the deterioration in results can be attributed to a significantly increased rate of comorbidity which has led to multiple increases in patients with American Society of Anesthesiologists (ASA) scores of 4–5 over the last several decades (2, 4).

These statistical trends have prompted a search for ways to improve patient outcomes. Advances in endoscopic technology have led to an increasing trend of laparoscopic surgery for PPUs worldwide. It is currently assumed that laparoscopic surgeries are either comparable to or substantially superior to open surgeries (1).

The number of systematic reviews (SRs) and meta-analyses in surgery is growing exponentially. However, heterogeneity, certain types of biases, and sparse data can cause misrepresentation of the combined results of primary studies in a pooled analysis. Meta-epidemiology, as a form of evidence synthesis, allows for the pooling of data and assessment of the diversity present in multiple and overlapping SRs (5, 6).

## Aim of the research

This study aimed to summarize evidence from systematic reviews of randomized controlled trials (RCTs) and reanalyze outcome data on laparoscopic suture repair of PPUs using trial sequential analysis.

## Materials and methods

There is no existing guidance on how to incorporate data excluded from primary studies into overviews of reviews appropriately (5). In the present meta-review, we used a meta-epidemiological approach and trial sequential analysis (TSA) that combined a conventional meta-analysis methodology with meta-analytic sample size considerations.

### Search strategy

Comprehensive search without language restriction was conducted by all the authors. The Cochrane Library, PubMed, Embase, MEDLINE, CINAHL, eLibrary, and ClinicalTrials.gov were searched before 1 June 2024 for all relevant systematic reviews.

An electronic search with filters designed for retrieving systematic reviews was conducted (7). A supplemental manual search for additional potentially relevant publications was subsequently conducted using citation searching and the “snowball” method. The result of the comprehensive systematic review search is presented in a PRISMA flow diagram.

### Inclusion criteria

For the meta-review, only SRs (with or without meta-analyses) of laparoscopic repair of PPUs were identified and considered. The study design of interest for the TSA was the RCTs included in the relevant SRs.

### Exclusion criteria

The exclusion criteria included studies of any design other than RCT, articles in a language other than English and systematic reviews without a statistical analysis of results.

### Data collection

The authors independently extracted data from the selected studies. The basic information about the characteristics of each systematic review included authors, year of publication, number of included studies, their design, and number of participants. Information about the RCTs included in the selected SRs (authors, years of research, country of research, and number of participants) was also extracted. We mapped primary study overlap within the included SRs as suggested by Pollock et al. (2023) (5).

### Assessment of methodological quality

Two meta-review authors (SP and TN) independently assessed the methodological quality of the selected SRs. Critical appraisal tools from the Oxford Centre for Evidence-Based Medicine were used (8). Information about the risk of bias of the RCTs contained within the relevant SRs was presented as narrative data.

### Statistical analysis

Copenhagen Trial Unit's software was used in the TSA to control for random errors and repetitive testing of the accumulated data.

TSA is a statistical tool designed to eliminate false positive conclusions (type I errors) and false negative conclusions (type II errors), providing more information on the precision and uncertainty of meta-analysis results. TSA is a methodology that combines the estimation of the total sample sizes of all included trials to meta-analyze the threshold for statistical significance (9).

For the determination of the sample size and to provide strong evidence of an intervention effect, the diversity-adjusted required information size (DARIS) was calculated. DARIS also allows one to set the boundaries of futility and indicate when a conclusion of no effect can be made conclusively before the required amount of information is reached (9).

We calculated the mean and standard deviation from the sample size and the median, range, or interquartile range for the meta-analytic reanalysis if necessary (10). The TSA was conducted according to the statistical methodology presented by Thorlund et al. (9). We estimated the diversity measure used to quantify heterogeneity (D^2^) and calculated the DARIS. The trial sequential monitoring boundaries were constructed on the basis of the DARIS using the Land and DeMets method (11). If the required sample size is reached, the boundaries of sequential monitoring or futility zone are crossed and further research may not be necessary.

The statistical computations of the TSA were performed by two co-authors (S.P. and T.N.). The type I (*α* risk) and type II (*β* risk) errors were set to 5% and 20%, respectively. We considered the control (laparotomy) group event proportion (Pc) as binary data and made an *a priori* estimate of a realistic intervention effect, expressed as a relative risk reduction (RRR) of 10%.

For continuous outcomes, the DARIS was calculated from an alpha error of 5%, a beta error of 20%, the estimated variance, and a minimal relevant difference (MIRD). Similar to published Cochrane Reviews, we considered the MIRD to be 1 cm for pain on a visual analog scale (VAS), 1 day for hospital stay, and 15 min for operative time (12, 13).

## Results

### Study selection, methodological quality, and results of SRs

**Figure 1:**
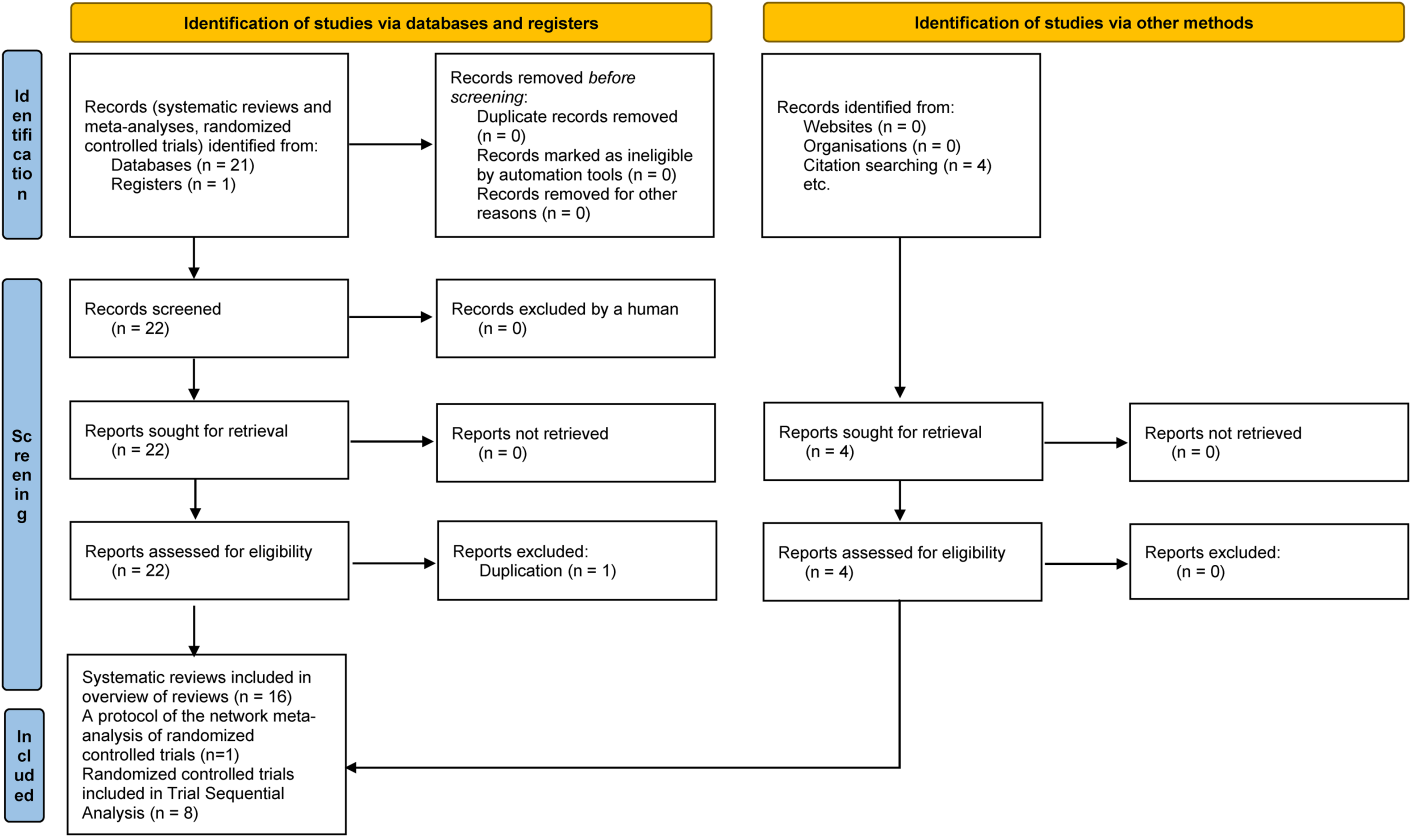
Stages of the comprehensive systematic review search.

In total, 16 relevant Cochrane and non-Cochrane SRs and a protocol of the network meta-analysis of RCTs that address laparoscopic repair of PPUs were identified and critically appraised (Figure 1, Tables 1, 2) (1, 14–29).

**Table 1:** Systematic reviews of laparoscopic repair for perforated peptic ulcer disease.

No.	Research groups (years)	Number of included studies (*n*)	Number of observations in meta-analyses (*n*)		
			Laparoscopic	Laparotomic	Total
Systematic reviews without meta-analyses					
1	Lee et al. (2020) (28)	5 RCTs, 18 NRSIs	N/A	N/A	25,531
Systematic reviews with meta-analyses of randomized controlled trials and non-randomized studies of interventions					
2	Lau (2004) (19)	2 RCTs, 11 NRSIs	294	364	658
3	Lunevicius and Morkevicius (2005) (20)	2 RCTs, 13 NRSIs	535	578	1,113
4	Zhou et al. (2015) (21)	5 RCTs, 24 NRSIs	1,890	3,378	5,268
5	Varcus et al. (2018) (22)	32 RCTs + NRSIs	3,488	5,208	8,696
6	Salman et al. (2022) (23)	7 RCTs, 38 NRSIs	N/A	N/A	8,456
7	Chan et al. (2023) (24)	4 RCTs, 25 NRSIs	1,687	3,624	5,311
8	Panin et al. (2023) (25)^a^	4 NRSIs	229	249^a^	478
9	Mohamedahmed et al. (2023) (26)^b^	4 NRSIs	438^b^	0	438
10	Li et al. (2023) (27)	29 RCTs + NRSIs	N/A	N/A	17,228
Systematic reviews with meta-analyses of randomized controlled trials					
11	Khatri et al. (2011) (14)	3 RCTs	141	128	269
12	Sanabria et al. (2015) (1)	3 RCTs	163	152	315
13	Antoniou et al. (2013) (15)	4 RCTs	151	138	289
14	Tan et al. (2016) (16)	5 RCTs	279	270	549
15	Cirocchi et al. (2018) (17)	8 RCTs	307	308	615
16	Quah et al. (2019) (18)	7 RCTs	319	312	631

RCT, randomized controlled trial; NRSI, non-randomized study of interventions; N/A, not available.

^a^ Laparoscopic vs laparoscopic assisted repair.

^b^ Laparoscopic repair with or without an omental patch.

**Table 2:** Main results of meta-analyses of randomized controlled trials and non-randomized studies of interventions.

No.	Research groups (years)	Main benefits of laparoscopic surgery
Systematic reviews without meta-analyses		
1	Lee et al. (2020) (28)	Unspecified due to study participants being described inconsistently in randomized clinical trials of peptic ulcer repair.
Systematic reviews with meta-analyses of randomized controlled trials and non-randomized studies of interventions		
2	Lau (2004) (19)	LR of PPUs confers superior short-term benefits in terms of postoperative pain and wound complications.
3	Lunevicius and Morkevicius (2005) (20)	Statistically significant findings in favor of LR were less analgesic use, shorter hospital stay, fewer SSI, and lower mortality rate.
4	Zhou et al. (2015) (21)	Earlier resumption of oral intake, shorter hospital stay, less analgesic use, fewer SSIs, and the reduced incidence of overall postop complications and hospital mortality for LR.
5	Varcus et al. (2018) (22)	LR showed similar or better results than OR in terms of morbidity, mortality, operation time, and hospital stay.
6	Salman et al. (2022) (23)	LR was associated with a shorter hospital stay and lower risks of mortality, septic shock, renal failure, and SSI.
7	Chan et al. (2023) (24)	Laparoscopic omental patch repair has lower mortality, overall morbidity, length of stay, intraoperative blood loss, and postoperative pain compared with open omental patch repair.
8	Panin et al. (2023) (25)	LRs are shorter and accompanied by a lower incidence of postoperative complications and shorter hospital stay compared with laparoscopy-assisted repair of PPUs.
9	Mohamedahmed et al. (2023) (26)	LR with or without an omental patch has comparable postoperative complications and mortality rates. Considering the shorter operative time, the no-omental patch approach is a more favorable choice.
10	Li et al. (2023) (27)	LR had a shorter postop hospital stay; less blood loss; fewer SSI, pneumonia, and respiratory complications; and lower postop morbidity and mortality rates.
Systematic reviews with meta-analyses of randomized controlled trials		
11	Khatri et al. (2011) (14)	LR was associated with lower pain score (SMD −1.14, 95% CI −2.15 to −0.12) and fewer complications (RR, 0.54; 95% CI 0.36 to 0.78).
12	Sanabria et al. (2015) (1)^a^	The results of LR were not clinically different from those of OR. Further RCTs are needed.
13	Antoniou et al. (2013) (15)	The results did not determine the comparative efficiency and safety of LR or OR repair of PPUs. Further RCTs are needed.
14	Tan et al. (2016) (16)^a^	The advantages of LR were lower SSI rate (OR: 0.32, 95% CI 0.17 to 0.62), shorter nasogastric tube duration (MD: −0.51, 95% CI −0.60 to −0.42), and less postop pain (SMD: −0.54, 95% CI: −0.88 to −0.19).
15	Cirocchi et al. (2018) (17)	This meta-analysis reported a significant advantage of LR only for postop pain in the first 24 h (−2.08; 95% CI −2.79 to −1.37) and for SSI (RR, 0.39; 95% CI 0.23 to 0.66).
16	Quah et al. (2019) (18)^a^	LR of PPUs has reduced morbidity (OR = 0.54, 95% CI: 0.37 to 0.79), SSI (OR = 0.3, 95% CI: 0.16 to 0.5), and total hospital stay.

PPU, perforated peptic ulcer; LR, laparoscopic repair; OR, open repair; SSI, surgical site infection; postop, postoperative; RCT, randomized controlled trial.

^a^ The meta-analysis included data on the suture-less closure of PPUs with fibrin sealant.

The aim of the planned network meta-analysis (29) was to compare surgical (including laparoscopic) and alternative approaches for the treatment of PPUs. The conclusions of the already published SRs were heterogeneous. Mismatches between systematic reviews' results were caused by substantial non-equivalence across included trial populations, different approaches to the application of laparoscopic repair of PPUs, and the various designs of the primary trials.

The main benefits of laparoscopic repair of PPUs according to the SRs that included results of RCTs and non-randomized studies of interventions (NRSIs) and the main conclusions of six meta-analyses that only polled the results of RCTs are presented in Table 2.

We mapped out the primary studies that were included in the systematic reviews of randomized controlled trials (30–38). Khatri et al. did 't specify which RCTs were included in their SR (14). Information about the amount of overlap between SRs is presented in Table 3.

**Table 3:** The primary studies contained within included systematic reviews of RCT (mapping of overlapping).

RCT (publication year)	Systematic reviews with meta-analyses of RCT				
	Sanabria et al. (2015) (1)^a^	Antoniou et al. (2013) (15)	Tan et al. (2016) (16)^a^	Cirocchi et al. (2018) (17)	Quah et al. (2019) (18)^a^
Lau et al. (1996) (30)	Included	Included	Included	Included	Included
Lau et al. (1998) (31)	Included	Included	—	Included	Included
Siu et al. (2002) (32)	Included	Included	Included	Included	Included
Bertleff et al. (2009) (33)	Included	Included	Included	Included	Included
Schietroma et al. (2013) (34)	—	—	Included	Included	Included
Shah et al. (2015) (35)	—	—	—	Included	Included
Zedan et al. (2015) (36)	—	—	—	Included	—
Ge et al. (2016) (37)	—	—	Included	Included	Included

Studies that chronologically could not be included in the review are marked in gray.

^a^ The meta-analysis included data on suture-less closure of PPUs with fibrin sealant.

Three overlapping reviews of RCTs met the inclusion criteria of the current meta-review and analyzed laparoscopic repair with sutures alone or in combination with an omental patch without fibrin sealant (14, 15, 17). There were no available data regarding the systematic review's methodology in the meta-analysis of Khatri et al. (14). The methodological quality of the systematic reviews of RCTs conducted by Antoniou et al. and Cirocchi et al. are presented in Table 4 according to the critical appraisal tools of the Oxford Centre for Evidence-Based Medicine.

**Table 4:** Critical appraisal sheet of the relevant systematic reviews.

	Antoniou et al. (2013) (15)	Cirocchi et al. (2018) (17)
What question (PICO) did the systematic review address?	Yes	Yes
Is it unlikely that important, relevant studies were missed?	Yes	Yes
Were the criteria used to select articles for inclusion appropriate?	Yes	Yes
Were the included studies sufficiently valid for the type of question asked?	Yes	Yes
Were the results similar from study to study?	No	No
What were the results?	Synthesis with and without meta-analyses	Synthesis with meta-analyses

### RCTs contained within the included systematic reviews

Since an additional TSA had been planned, we summarized the information of all the published RCTs included in the relevant SRs which are presented in Table 5.

**Table 5:** General information about the randomized controlled trials of laparoscopic repair of perforated peptic ulcers.

	Research groups (years)	Country	Surgery	
			Laparoscopic	Laparotomic
1	Lau et al. (1992–1994)^a^	Hong Kong, China	*n* = 24	*n* = 21
2	Lau et al. (1995–1996)	Hong Kong, China	*n* = 9	*n* = 10
3	Siu et al. (1994–1997)	Hong Kong, China	*n* = 63	*n* = 58
4	Bertleff et al. (1999–2005)	Netherlands	*n* = 52	*n* = 49
5	Schietroma et al. (2005–2011)	Italy	*n* = 57	*n* = 58
6	Shah et al. (2009–2011)	India	*n* = 25	*n* = 25
7	Zedan et al. (2012–2014)	Egypt	*n* = 21	*n* = 24
8	Ge et al. (2010–2014)^b^ Wang et al. (2010–2014)^b^	China	*n* = 58	*n* = 61
Total			*N* = 309	*N* = 306

^a^ Without data about laparoscopic suture-less repair.

^b^ Publications based on the results of one RCT.

An evaluation of the domain-specific risk of bias of each RCT included in the relevant SRs was already conducted by the systematic review authors. There was a high risk of performance and detection bias due to a lack of blinding. Blinding was not possible as the RCTs compared open surgery with laparoscopy (1, 16).

### Synthesis of results

As there were no supplements to the existing primary study evaluations, in the next step of the meta-review, we extracted the outcome data reported in each of the included SRs (Table 6).

**Table 6:** Summarized outcome data of systematic reviews.

	Effect estimate		
	Khatri et al. (2011) (14)	Antoniou et al. (2013) (15)	Cirocchi et al. (2018) (17)
VAS scores (24 h)	SMD = 1.14 (−2.15 to −0.12)^a^ *(the time period is not specified)*	Favor laparoscopic *(narratively reported data)*	MD = −2.08 (−2.79 to −1.37), *I*^2^ = 69%
VAS scores (72 h)		NR	Data were not used in the meta-analysis.
Operative time (min)	SMD = 0.53 (−0.47 to 1.54)	WMD = 0.38 (1.22 to 1.99)	MD = −5.10 (−7.38 to −2.82), *I*^2^ = 69%
Hospital stay	SMD = −0.15 (−0.38 to 0.09)	Favor laparoscopic (*narratively reported data)*	MD = −2.32 (−5.17 to 0.53), *I*^2^ = 89%
Mortality	RR = 0.40 (0.11 to 1.36)	OR = 0.36 (0.10 to 1.32)	RR = 0.45 (0.16 to 1.28), *I*^2^ = 0%
Morbidity	RR = 0.54 (0.36 to 0.78)	Meta-analysis of “major” complications	Meta-analyses of some selected complications
Intra-abdominal abscess	NR	NR	RR = 1.50 (0.43 to 5.23), *I*^2^ = 32%
Surgical site infection	NR	NR	RR = 0.39 (0.23 to 0.66), *I*^2^ = 0%

NR, not reported; MD/SMD, mean difference/standardized mean difference; WMD, weighted mean difference; RR, relative risk; OR, odds ratio; *I*^2^, quantification of heterogeneity as the inconsistency.

^a^ 95% confidence interval.

There was no significant difference between laparoscopic and open repair in terms of mortality. However, the results of all meta-analyses favor laparoscopic surgery with regard to less postoperative pain and enhanced recovery (14, 15, 17). One meta-analysis also noted a reduction in the incidence of postoperative wound complications (17).

### Trial sequential analysis

The reanalysis of the meta-analytic findings and adjustments of sample size by the TSA confirmed that laparoscopic repair was associated with less postoperative pain. The inferences about statistical significance were confirmed by the strength of the evidence (Table 7, Supplementary Figures S2А,B) and future trials are unlikely to change them.

**Table 7:** Reanalysis of meta-analytic findings with additional values from the trial sequential analysis.

	Meta-analyses				TSA values				
	Number of studies (references)	Current number of participants	P_E_ (range)	P_C_ (range)	Measures of treatment effect^a^	D^2^	Scenario	DARIS (*n*)	% of the required size
Day 1 (VAS scores)	4 RCTs (31, 32, 33, 35)	312	3–4.4	5–7	MD = −1.95 (−2.9 to −1.1)^b^	89%	*α* = 5%, *β* = 20%, MIRD = 1	536	58.2
Day 3 (VAS scores)	2 RCTs (32, 33)	222	1.6–3.8	5–13.8	MD = −1.36 (−2.1 to −0.6)^b^	81%	*α* = 5%, *β* = 20%, MIRD = 1	277	80.1
Operative time (min)	7 RCTs (30, 32–37)	596	42–113	35–110	MD = 7.96 (−9.1 to 24.4)^b^	97%	*α* = 5%, *β* = 20%, MIRD = 15	1,487	40.1
Hospital stay (days)	7 RCTs (30, 32–37)	596	3–8.1	5–13.8	MD = −2.45 (−4.3,−0.62)^b^	92%	*α* = 5%, *β* = 20%, MIRD = 1	4,084	14.6
Mortality (Y/N)	8 RCTs (30–37)	615	4/218^c^ (1.83%)	10/213^c^ (4.69%)	RR = 0.45 (0.16 to 1.28)^b^	0%	*α* = 5%, *β* = 20%, Pc = 4.69%, RRR = 10%	60,497	0.71^d^
Intra-abdominal abscess (Y/N)	7 RCTs (30, 32–37)	596	7/300 (2.3%)	14/296 (4.7%)	RR = 0.58 (0.18 to 1.84)^b^	0%	*α* = 5%, *β* = 20%, Pc = 4.7%, RRR = 10%	83,100	0.57
Surgical site infection (Y/N)	7 RCTs (30, 32–37)	596	16/300 (5.3%)	43/296 (14.5%)	RR = 0.38 (0.22 to 0.64)^b^	0%	*α* = 5%, *β* = 20%, Pc = 14.5%, RRR = 10%	17,737	3.36

VAS, visual analog score; MD, mean difference; MIRD, minimal relevant difference; RCT, randomized controlled trials; P_E_, the proportion with an outcome in the intervention (laparoscopic) group; P_C_, the proportion with an outcome in the control (laparotomic) group; RR, relative risk; D^2^, diversity; RRR, relative risk reduction; α, α risk; β, β risk; DARIS, diversity-adjusted required information size.

^a^ Random-effect model.

^b^ 95% confidence interval.

^c^ Trials with zero events were ignored.

^d^ The TSA ignored some trials due to lack of events.

The calculated DARIS for operative time and hospital stay were 40.1% and 14.6%, respectively, and the TSA showed neither significant benefit nor harm of laparoscopic surgery in the attained information size in the meta-analysis (Table 7, Supplementary Figures S3A,B).

Further trials with regard to mortality, surgical site infection, and intra-abdominal abscesses are not very promising because the DARIS did not exceed 5% after the combined results of all the available RCTs. Thus, the main significant results include a reduction in both pain on postoperative day 1 and the duration of the procedure (Table 7, Supplementary Figures 4A–C).

## Discussion

A perforated peptic ulcer is a common surgical condition. The results of treatment of certain groups of patients, e.g., comorbid patients, remain unsatisfactory. This is despite obvious advances in surgical and anesthetic techniques and intensive care approaches. One direction for improving treatment outcomes appears to be the introduction of evidence-based techniques and practices (1). Clinical guideline recommendations are based on the combined results of randomized trials in meta-analyses.

This study found only 16 relevant Cochrane and non-Cochrane SRs that address laparoscopic repair of PPUs (1, 14–29). Their conclusions were heterogeneous due to a substantial non-equivalence across trial populations, different approaches of laparoscopic repair for PPU, and various designs.

Only three reviews of overlapping RCTs met the inclusion criteria of the meta-review and analyzed laparoscopic repair with sutures alone or in combination with an omental patch (14, 15, 17). Despite the popular opinion of experts about the advantages of laparoscopy, no differences were reported in most of the clinical outcomes. The only access-related significant difference was in lower postoperative pain scores after laparoscopic repair on day 2 (according to data from two RCTs). This also was the only parameter that achieved 82% of the DARIS. Hence, subsequent studies are unlikely to change this conclusion.

For the other evaluation criteria, the DARIS scores ranged from 0.57% (intra-abdominal abscess) to 58.2% (pain on postoperative day 1). The calculated DARIS for operative time and hospital stay were 40.1% and 14.6%, respectively, and the TSA showed neither significant benefit nor harm of laparoscopic surgery in the attained information size in the meta-analysis. Further trials with regard to mortality, surgical site infection, and intra-abdominal abscesses are not very promising because the DARIS did not exceed 5% after combining the results of eight RCTs.

To our knowledge, this is the first study of laparoscopic suture repair of perforated peptic ulcer disease that summarizes evidence from systematic reviews in the format of meta-review with trial sequential analysis.

There remains a lack of high-quality evidence-based research. Over the past three decades, only eight RCTs have been conducted worldwide (1, 17). At the same time, it must be stated that the DARIS will not be achieved in the foreseeable future in terms of the main indicator of treatment effectiveness—mortality rate.

Future research should explore metadata on issues where a TSA is available and the DARIS is achievable. It seems important to focus on the efficacy and safety of minimally invasive PPU treatment technologies in subgroups of patients with comorbidity or immunosuppression, in the elderly, etc.

## Conclusion

Summarization of evidence from systematic reviews and reanalysis using TSA confirmed sufficient evidence for one postoperative outcome. Laparoscopic suture repair of PPUs is accompanied by lower pain scores at 24–72 h compared with open repair, improving postoperative recovery. On the issue of postoperative complications and mortality, achieving a DARIS through additional studies seems unpromising.

## Data Availability

The original contributions presented in the study are included in the article/Supplementary Material, further inquiries can be directed to the corresponding author.
