# Supplementary figures and images for: Laparoscopic suture repair for perforated peptic ulcer disease: a meta-review and trial sequential analysis
Source: Front Surg. 2025 Feb 12;12:1496192. doi: 10.3389/fsurg.2025.1496192 (PMC11861353; doi:10.3389/fsurg.2025.1496192)

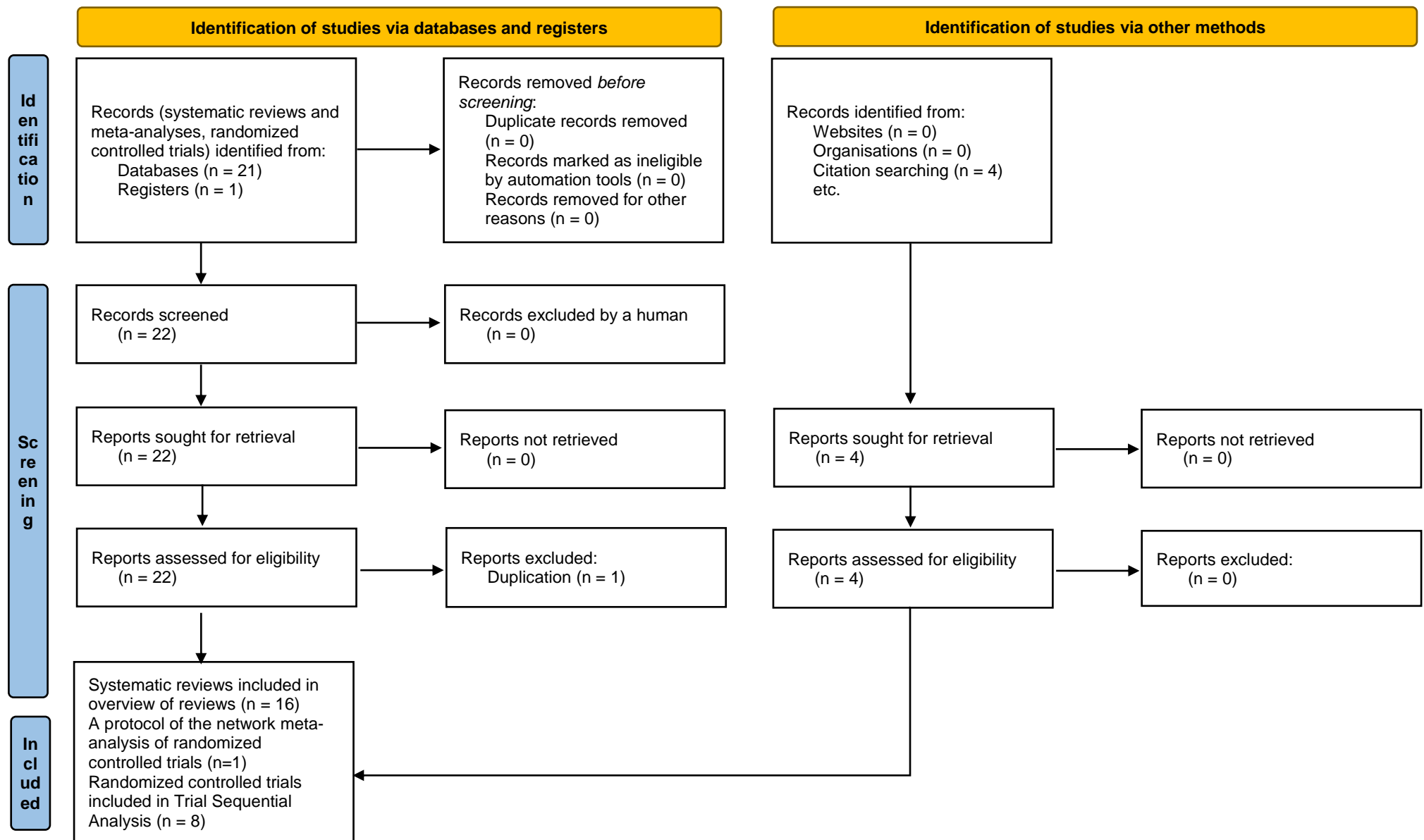

FIGURE 1. Stages of comprehensive systematic review search

Supplement: Supplementary file 1 [file Datasheet1.pdf]
