# Supplementary material for: Laparoscopic suture repair for perforated peptic ulcer disease: a meta-review and trial sequential analysis
Source: Front Surg. 2025 Feb 12;12:1496192. doi: 10.3389/fsurg.2025.1496192 (PMC11861353; doi:10.3389/fsurg.2025.1496192)
Supplement: Supplementary file 2 [file Datasheet2.pdf]

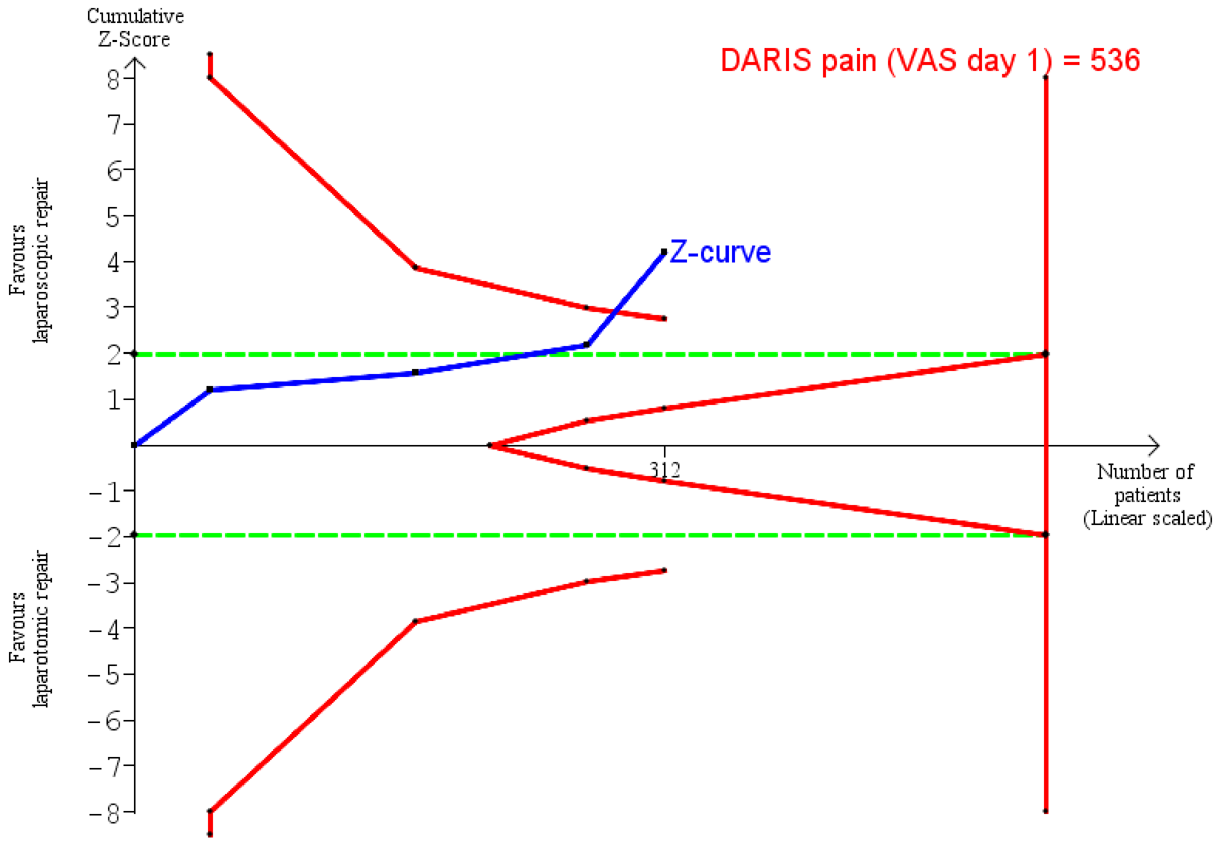

Supplemental Figure 2A: Trial sequential analysis of pain (VAS day 1).

TSA performed according to the data of the meta-analysis summarized in the table 7. The DerSimonian-Laird method and the constant continuity correction method (the sum of two correction factors is 1.0) were used for the TSA. The diversity-adjusted required information size (DARIS) of 536 participants was calculated based on a minimal relevant difference of a minimal relevant difference of 1 cm on the visual analogue scale, alpha 5%, beta 20% (giving power of 80%), a variance of 1.93 and heterogeneity correction with diversity (D^2) of 89%.

Trial sequential analysis showing that the cumulative Z-curve crosses the trial sequential monitoring boundary after the third trial. Although the DARIS has not been reached, the findings are consistent with laparoscopic repair decreasing pain within 24 hours compared with laparotomic surgery.
