# Supplementary material for: Laparoscopic suture repair for perforated peptic ulcer disease: a meta-review and trial sequential analysis
Source: Front Surg. 2025 Feb 12;12:1496192. doi: 10.3389/fsurg.2025.1496192 (PMC11861353; doi:10.3389/fsurg.2025.1496192)
Supplement: Supplementary file 3 [file Datasheet3.pdf]

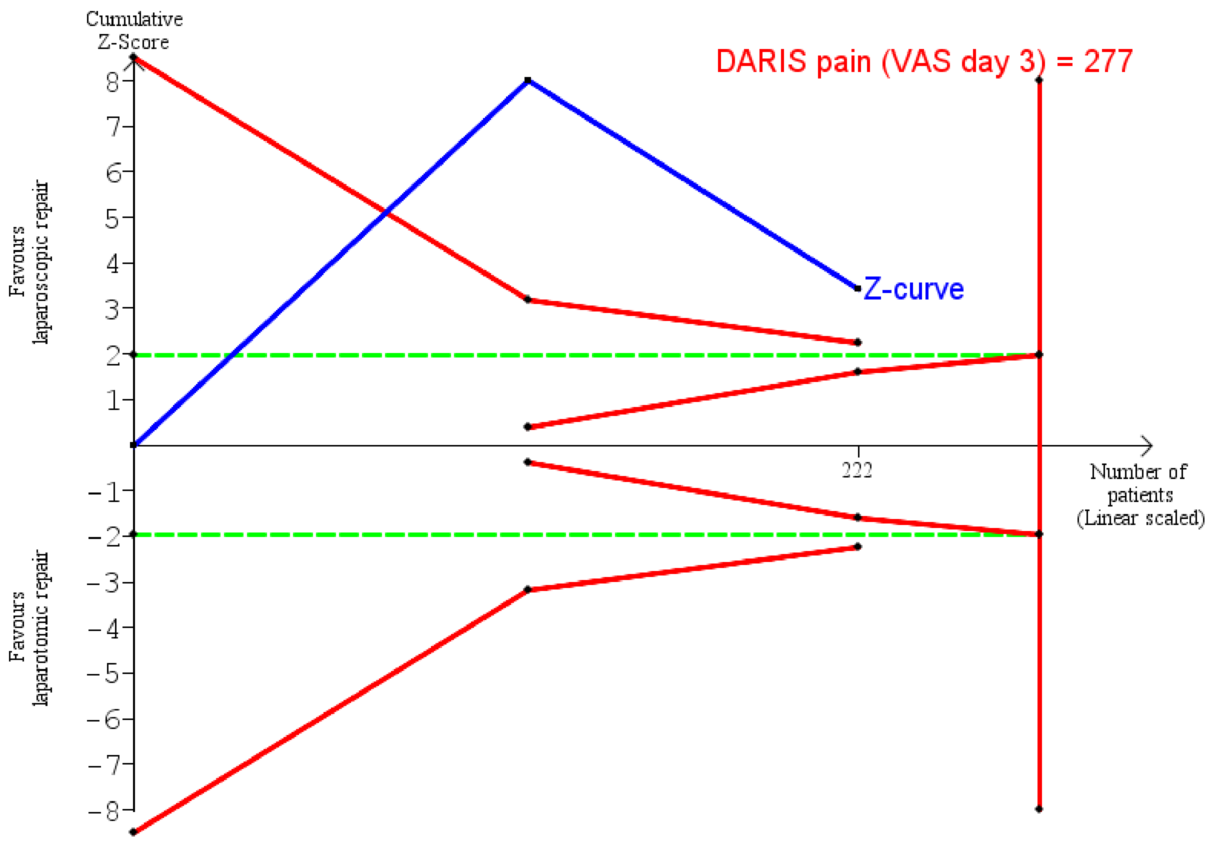


Supplemental Figure 2B: Trial sequential analysis of pain (VAS day 3).

TSA performed according to the data of the meta-analysis summarized in the table 7. The DerSimonian-Laird method and the constant continuity correction method (the sum of two correction factors is 1.0) were used for the TSA. The diversity-adjusted required information size (DARIS) of 277 participants was calculated based on a minimal relevant difference of 1 cm on the visual analogue scale, alpha 5%, beta 20% (giving power of 80%), a variance of 1.69 and heterogeneity correction with diversity ($D^2$) of 81%.

Trial sequential analysis showing that the cumulative Z-curve crosses both the conventional boundary $\pm$1.96 and the trial sequential monitoring boundary. The results are compatible with benefits of laparoscopic repair for perforated peptic ulcer.
