# Supplementary material for: Laparoscopic suture repair for perforated peptic ulcer disease: a meta-review and trial sequential analysis
Source: Front Surg. 2025 Feb 12;12:1496192. doi: 10.3389/fsurg.2025.1496192 (PMC11861353; doi:10.3389/fsurg.2025.1496192)
Supplement: Supplementary file 4 [file Datasheet4.pdf]

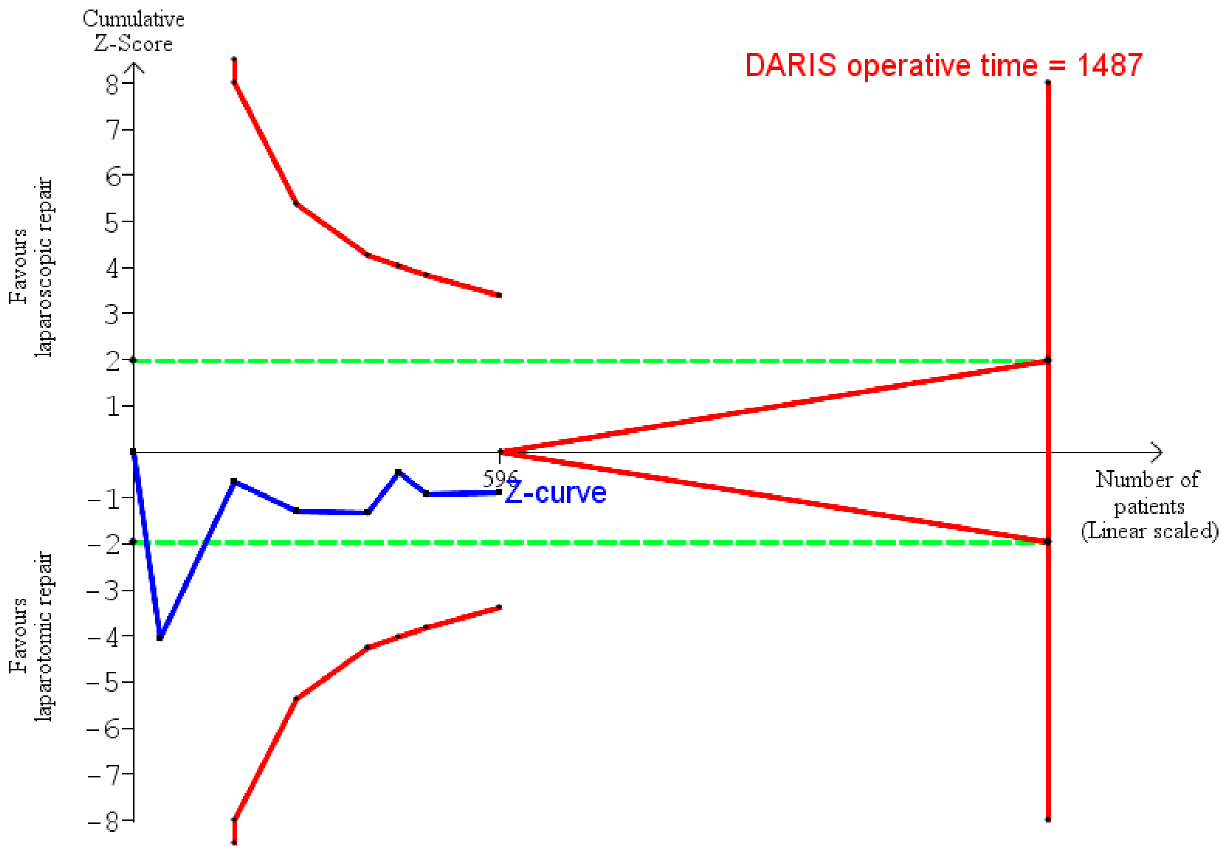

Supplemental Figure 3A: Trial sequential analysis of operative time.

TSA performed according to the data of the meta-analysis summarized in the table 7. The DerSimonian-Laird method was used for the TSA. The diversity-adjusted required information size (DARIS) of 1487 participants was calculated based on a minimal relevant difference of 15 minutes, alpha 5%, beta 20% (giving power of 80%), a variance of 280.46 and heterogeneity correction with diversity (D^2) of 97%.

After accruing 596 participants in the seven trials, only 40.1% of the DARIS has been reached. Although, the conventional boundary $\pm$1.96 was initially crossed for harm, Z-curve is located in the area of potentially false neutral results.
