# Supplementary material for: Laparoscopic suture repair for perforated peptic ulcer disease: a meta-review and trial sequential analysis
Source: Front Surg. 2025 Feb 12;12:1496192. doi: 10.3389/fsurg.2025.1496192 (PMC11861353; doi:10.3389/fsurg.2025.1496192)
Supplement: Supplementary file 6 [file Datasheet6.pdf]

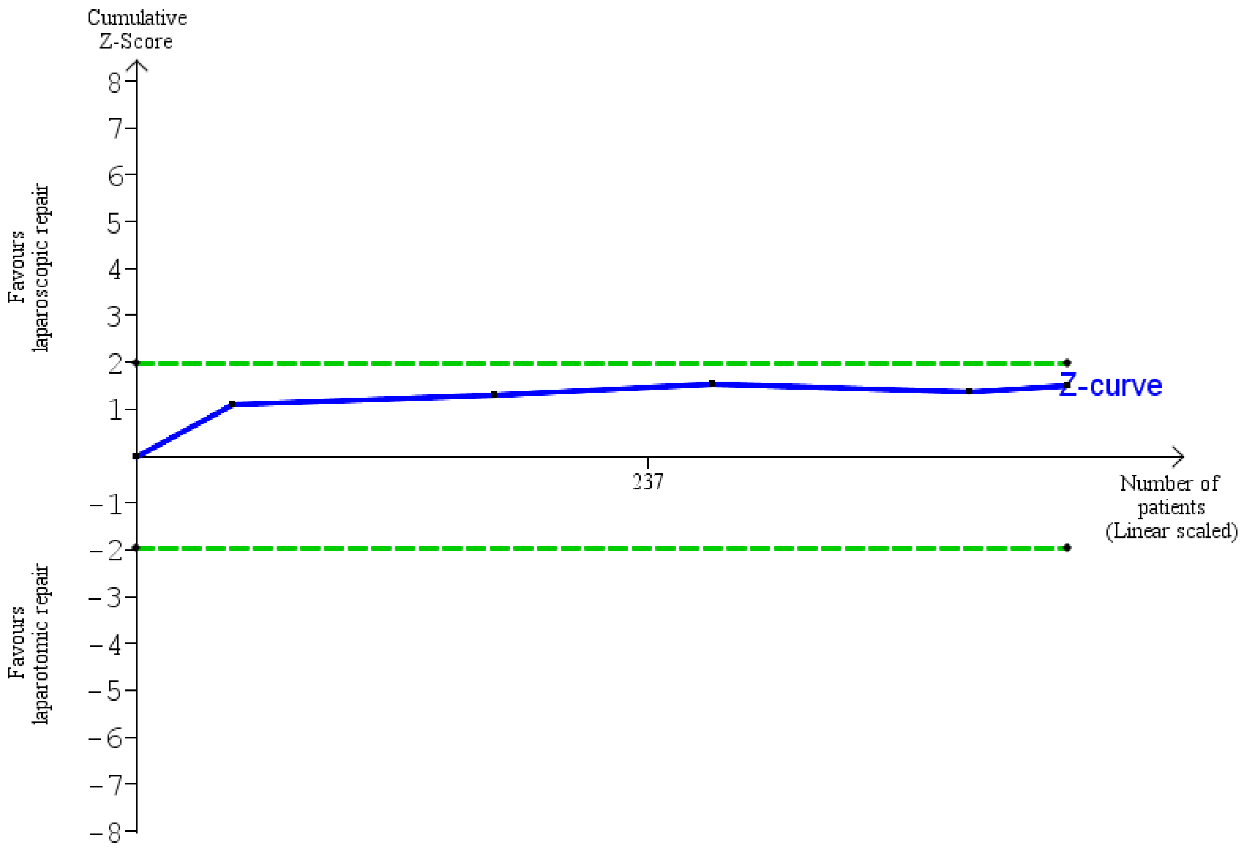

Supplemental Figure 4A: Trial sequential analysis of mortality.

The diversity-adjusted required information size (DARIS) of 60497 participants was calculated based on the proportion with an outcome in the control group (Pc) 4.69%, relative risk reduction (RRR) 10%, alpha 5%, beta 20% (giving power of 80%) and a diversity (D2) of 0%. The DerSimonian-Laird method and the constant continuity correction method (the sum of two correction factors is 1.0) were used for the TSA.

After accruing 431 participants in eight trials, only 0.71% of the DARIS has been reached. Accordingly, the trial sequential analysis program does not show neither the futility area nor the trial sequential boundaries due to small attained information size.
