# Supplementary material for: Laparoscopic suture repair for perforated peptic ulcer disease: a meta-review and trial sequential analysis
Source: Front Surg. 2025 Feb 12;12:1496192. doi: 10.3389/fsurg.2025.1496192 (PMC11861353; doi:10.3389/fsurg.2025.1496192)
Supplement: Supplementary file 7 [file Datasheet7.pdf]

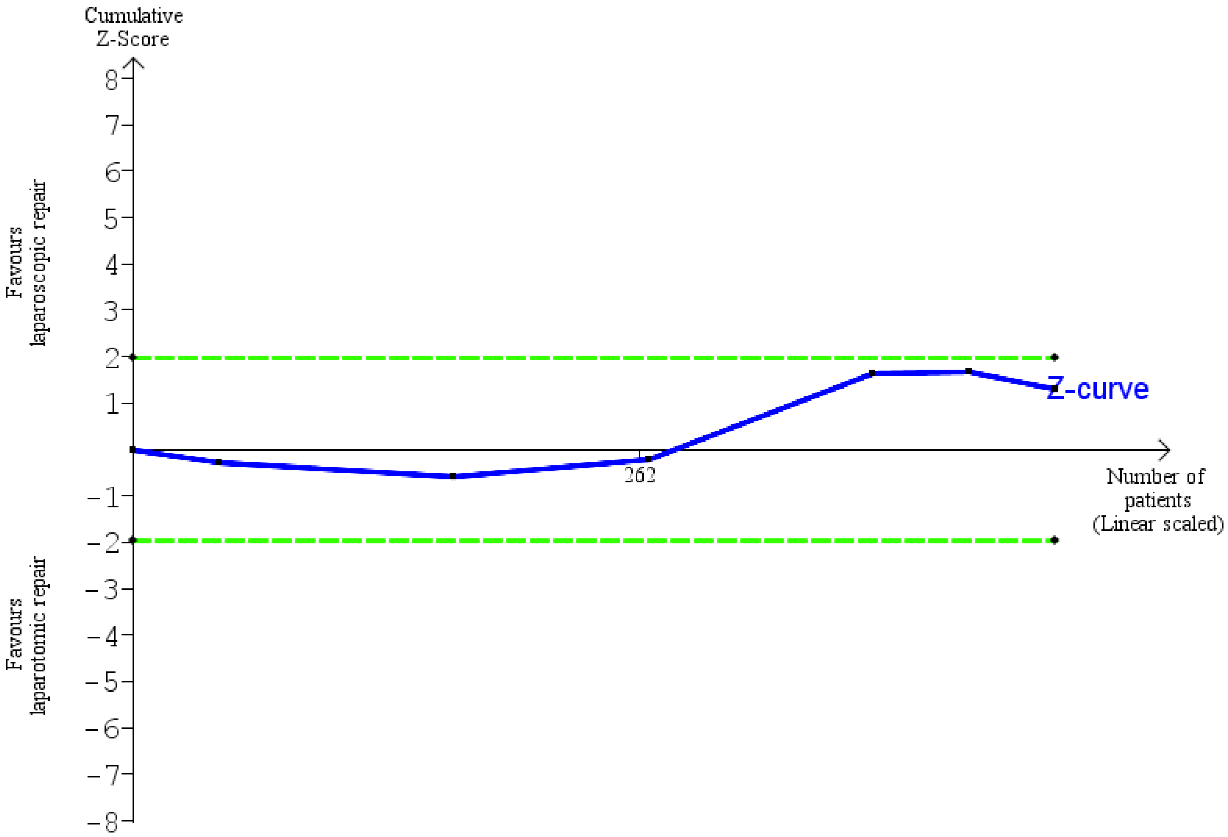

Supplemental Figure 4B: Trial sequential analysis of Intra-abdominal abscess.

The diversity-adjusted required information size (DARIS) of 83100 participants was calculated based on the proportion with an outcome in the control laparotomic group (Pc) 4.7%, relative risk reduction (RRR) 10%, alpha 5%, beta 20% (giving power of 80%) and a diversity (D2) of 0%. The DerSimonian-Laird method and the constant continuity correction method (the sum of two correction factors is 1.0) were used for the TSA.

After accruing 596 participants in seven trials, only 0.57% of the DARIS has been reached. The conventional boundaries have not been crossed by the cumulative Z-curve. The trial sequential monitoring boundaries and the futility area have not been drawn on the graphs due to small attained information size.
